# Update on Biomarkers to Monitor Clinical Efficacy Response During and Post Treatment in Allergen Immunotherapy

**DOI:** 10.1007/s40521-017-0117-5

**Published:** 2017-03-10

**Authors:** Lubna Kouser, Jasper Kappen, Ross P. Walton, Mohamed H. Shamji

**Affiliations:** 1 Immunomodulation and Tolerance Group, Allergy and Clinical Immunology, Inflammation, Repair and Development, Faculty of Medicine, Imperial College London, National Heart and Lung Institute, London, SW7 2AZ UK; 2 Department of Pulmonology, STZ centre of excellence for Asthma & COPD, Sint Franciscus Vlietland group, Kleiweg 500, 3045 PM Rotterdam, The Netherlands; 3 Airway Disease Infection Section, Imperial College London, part of the Medical Research Council and Asthma UK Centre for Allergic Mechanisms of Asthma, St. Mary’s Hospital, National Heart and Lung Institute (NHLI), London, W2 1PG UK

**Keywords:** Allergen immunotherapy, Tregs, ILC2, Biomarkers, IgG4, AIT

## Abstract

Allergen immunotherapy (AIT) is an immune modulating treatment for allergic diseases. Although highly effective, some patients do not respond to the treatment. To date there are no surrogate biomarkers that are predictive of the clinical response to AIT. More and more is known about the underlying immunological mechanism involved in AIT. Through modulation of both innate and adaptive immune responses, involving reduced ILC2 and enhanced Treg and Breg induction and functionality, along with induction of IgG4 antibody production which have the capacity to inhibit both allergen-induced basophil responsiveness and CD23-mediated IgE-facilitated allergen presentation, the result is an immune skewing towards a more balanced Type I response. So far, however there is not a clear correlation with the observed immunological changes and predictive correlates of clinical efficacy. The most promising biomarker of successful AIT is IgE-FAB as a reflection of functional IgG4. Cellular responses and cytokine analysis gives a great deal of insight into the mechanisms of AIT but may not represent useful or indeed reliable biomarkers in a clinical setting. There is a need for more research for confirmation and interpretation of the possible association with biomarkers and clinical response to AIT.

## Introduction

Allergen immunotherapy (AIT) is the only disease-modifying therapy available for IgE-mediated diseases such as allergic rhinitis, allergic asthma and atopic dermatitis[1, 2]. AIT is a safe and effective treatment indicated only in those patients whose symptoms are mostly uncontrolled by conventional pharmacotherapy such as antihistamines or nasal steroids [3, 4]. AIT, which can be administered either subcutaneously (SCIT) or sublingually (SLIT), reduces both symptoms and the need of rescue medication [5–7], improves patient’s quality of life [8] and confers long-term clinical benefits after cessation of treatment [9, 10••, 11, 12, 13•]. Although AIT is effective, the degree of remission depends on several unidentified factors (1, 6, 13•, 14–17). It is therefore, essential to determine biomarkers that would: identify those patients most likely to respond to therapy; indicate when to stop treatment; predict symptomatic relapse and inform on when to perform a booster AIT. Application of such knowledge, would undoubtedly contribute to the use of biomarkers of AIT in personalised medicine [18]. Novel insight in to the mechanism which govern AIT is essential for the identification of robust biomarkers. This review presents an updated overview of the underlying mechanism and novel potential biomarkers of AIT.

## Mechanisms of AIT

The allergic response cascade is characterized by a complex network of dysregulated immunological events. Through the administration of high allergen dose during AIT, both innate and adaptive immune responses are modulated. Efficacy of, and tolerance induction through AIT has been shown to be associated with decreased numbers of infiltrating mast cells [19, 20•], basophils [21], and eosinophils in the nasal mucosa [20•, 22], in addition to a reduction in the frequency of group 2 innate lymphoid cells (ILC2) [23••] and type 2 T helper (Th2) cells [24, 25•], in the periphery. AIT modulates dendritic cell responses (DCs) [26] which leads to immune deviation from T helper type 2 to type 1 response, induction of interleukin (IL)-10+, IL-35+, TGF-β+ and generation of FoxP3+ regulatory T cells. Moreover, IL-10+ B regulatory cells and IgG4 antibodies responses during and after cessation of AIT have been observed [27–31]. These antibodies have the capacity to inhibit both allergen-induced basophil responsiveness and CD23-mediated IgE-facilitated allergen presentation [10••]. Understanding the mechanisms of AIT is essential as it paves the way to identify novel determinant biomarkers of AIT success and therapeutic targets which when combined with AIT could potentially restore an immune tolerance state.

## Immunomodulation of ILC2s and IL13^+^ ILC2s by AIT

**Fig. 1:**
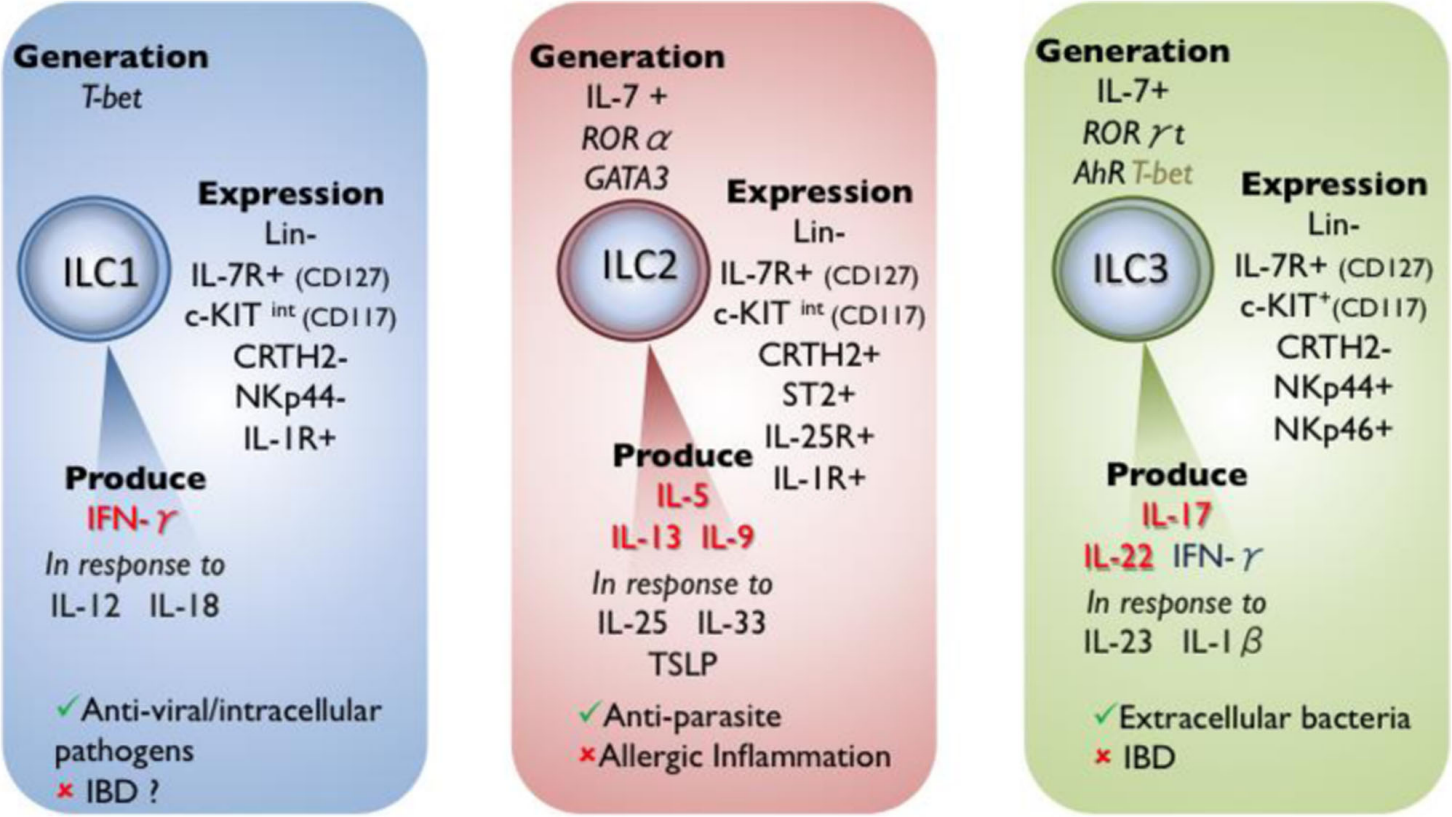
Innate lymphoid cells. Three groups of non-cytotoxic lineage negative ILCs are defined as ILC1, ILC2, and ILC3. ILCs express the subunits of cytokine receptors such as interleukin (IL)-2 receptor-α (CD25) and IL-7 receptor-α (CD127) but do not express the somatically rearranged antigen receptors as T and B cells and lack antigen specificity. Thus exhibit functions in an antigen-independent manner. ILC1s produce IFN-γ in response to IL-12 and IL-18 providing immunity to intracellular bacteria and parasites. Whereas ILC2s are stimulated by IL-25, IL-33, or TSLP to produce Th2 cytokines such as IL-5, IL-13, and IL-9 causing allergic inflammation. ILC2s also provide immunity to helminths as well as tissue repair. The ILC3s are driven by IL-23, IL-1β to produce IL-17, IL-22, and IFN-γ, which promote immunity to extracellular bacteria and tissue repair.

Innate lymphoid cells (ILCs) are morphologically similar to lymphocytes but lack the rearranging antigen receptors [32]. ILCs can be grouped into three known subsets: ILC1, ILC2, and ILC3 (Fig. 1), which is defined by a combination of surface markers, transcription factors that are critical for their generation, and cytokines that they produce [33].

In particular, ILC2 effector function is driven by epithelial derived mediators IL-33 [34], IL-25 [35], TSLP [36], as well as leukotriene D4 to produce Th2 cytokines such as IL-4, IL-5, IL-9, and IL-13 [33]. ILC2s promote type 2 allergic inflammation, and tissue repair [33]. First described in mouse, an analogous population of ILC2 was identified in human skin, revealing a population of Lin^−^ CD25^+^ ST2^+^ c-Kit^+^ CD127^+^ ICOS^+^ which did not express markers of ILC3, CD4, NKp46, and RORɣt [36]. Shortly after, the presence of ILC2s, which shared the same morphology as those in the skin, was confirmed in other organs [37]. Skin-specific expression of IL-33 in transgenic mice was associated with atopic dermatitis like cutaneous appearance. These mice had increased levels of Lin^−^ST2^+^ Sca-1^+^ ILC2s in the skin lesions, peripheral blood, and regional lymph nodes. Lin^−^ST2^+^ Sca-1^+^ produced IL-5 and IL-13 in response to IL-33 compared to wild-type mice that did not produce this effect in the lymph nodes, suggesting that IL-33 stimulates ILC2s [34]. However, Kim and Colleagues [36] reported that the ILC2s were induced by TSLP in an IL-33 independent manner. It was further elucidated that the response of ILC2s to TSLP, IL-25, or IL-33 regulated skin inflammation. High levels of ILC2s were found in the skin of atopic dermatitis with the elevated expression of IL-17RB (IL-25R), ST2 (IL-33R), and TSLP receptors. The increased expression was associated with elevated levels of IL-25 and IL-33 expression. BALB/c strain mice deficient in cytokine receptors for IL-33, IL-25, and TSLP had decreased skin inflammation of ear tissue and skin-draining lymph nodes; however in response to TSLP, the reduction in skin inflammation was moderate. These findings confirmed these cytokines are involved in the regulation of ILC2s in the pathogenesis of atopic dermatitis, an IgE-mediated disease [37].

The relevance of ILC2s to the pathophysiology of allergic rhinitis was firstly demonstrated in subjects who underwent intranasal cat allergen provocation [38•]. Nasal cat allergen challenge resulted in an increase of peripheral blood ILC2s expressing CD84. Diluent challenge resulted in no change in the percentage of ILC2s whereas the levels of ILC2s were promptly elevated after 4 h when challenged with cat allergen. These findings indicated that acute induction of ILC2s could play a role in the pathogenesis of allergic rhinitis [38•]. Moreover, peripheral blood CD117^+^ILC2s and IL-13^+^ILC2s have been shown to be increased in grass pollen allergic individuals with seasonal allergic rhinitis following natural grass pollen allergen exposure during the pollen season compared to out of the pollen season [23••]. Grass pollen SCIT blunted the seasonal increases of CD117^+^ILC2s as well as IL-13^+^ILC2s [23••]. Interestingly, the proportion of ILC2 correlated with visual analog scores in allergic and treated individuals during the pollen season [23••]. It is important to note that outside the pollen season, the frequency of CD117^+^ILC2s and IL-13^+^ILC2s between grass pollen allergics and non-allergic controls remained unaltered [23••]. These findings were confirmed by recent reports that demonstrated that the number of ILC2s found in the peripheral blood of allergic subjects were similar to the non-allergics [39]. However, allergic asthmatics had elevated numbers of ILC2s and ILC3s in the periphery. This study also confirmed that the frequency of ILC2s and ILC3s were high during the grass pollen season in those that were sensitized to grass pollen allergen. In addition, a short 4-month SLIT did not reduce the frequency of ILC2s. It is likely that the discrepancy of findings between the SCIT and SLIT study was due to the enumeration of ILC2s in active and placebo groups being conducted outside of the pollen season [39]. More studies are needed to evaluate the effect of AIT on ILC2s and whether there is a relationship between ILC2s and clinical response to AIT.

## Molecular markers of DCs as biomarkers AIT success

Dendritic cells are the key orchestrators of both the innate and the adaptive immune responses, one aspect of which includes regulation of T cell responses. When triggered by an allergen, immature DCs polarize into DC1s, DC2s, DC17s, or DCregs, which in turn can differentiate T cells into Th1 cells (DC1s), Th2 cells (DC2s), Th17 cells (DC17s), or regulatory T cells (DCregs) [40••]. Gueguen and colleagues showed that DCregs or tolerogenic DCs were generated when DCs were exposed to dexamethasone (DEX), which resulted in an increased expression of Ig-like transcript 2 (ILT2) and Ig-like transcript 4 (ILT4). Co-culture of tolerogenic DCs with CD4^+^ T cells resulted in upregulation of IL-10 but not Foxp3. Moreover, DC1 and DC17 effector DC markers such as CD71, FSCN1, IRF4, NMES1, MX1, and TRAF1 were significantly upregulated. Interestingly, tolerogenic DC markers were associated with ANXA1, complement component 1 (C1Q), CATC, GILZ, F13A, FKBP5, Stabilin-1 (STAB1), and TPP1 molecules. ANXA1 and FKBP5 were overexpressed in regulatory DCs. The expression of C1Q A, B, and C, as well as STAB1 was increased in peripheral blood of monocyte derived tolerogenic DCs in subjects of who received sublingual grass pollen immunotherapy. C1QA, C1QB, C1QC, and STAB1 were also found to be upregulated in patients with confirmed clinical response to treatment. The C1Q and STAB1 expression was consistently induced in response to treatment and also correlated with clinical benefit in SLIT-treated patients [41]. Gueguen and colleagues identified additional molecular markers that are differentially regulated in DC2s and DCregs, evaluated their role as predictive biomarkers of efficacy. Changes in expression of 5 combined DCreg/DC2-associated markers in PBMCS correlated with clinical efficacy of SLIT at 2 and 4 months. Interestingly, the four markers of DC2 cells (CD141, GATA3, OX40L, and RIPK4) were decreased after 4 months of AIT in allergic rhinitis patients, whereas the expression of 4 DCreg cell markers (i.e., C1QA, FcεRIIIA, FTL, and SLCO2B1) were increased in the peripheral blood of allergic rhinitics. This implies that AIT stimulates the expression of markers for DC2 and DCreg cells associated with clinical efficacy [40••]. After 2 and 4 months of AIT, the optimal combination of five molecular markers of which three DC2 (CD141, GATA3, and RIPK4) and two DCreg (C1QA and FcγRIIIA) resulted in effective classification of clinical responders from non-responders. The combined markers had a sensitivity of 90.48% and a specificity of 61.9%. Although, these findings were very interesting, they need to be validated in large clinical studies of SCIT, SLIT and then in clinical practice.

## T cell responses and AIT

Inflammatory responses in allergic diseases are widely considered to be Th2 mediated. Th2 cytokines such as IL-4, IL-5, and IL-13 subsequently are responsible for the induction of effector cells [42]. The production of specific IgE by B cells is also facilitated by Th2. AIT is associated with immune deviation from Th2 to Th1 responses and induction of regulatory T cells. Regulatory T cells (Tregs) modulate the immune response, mediate immune tolerance, and prevent autoimmune diseases [43]. A substantial body of evidence also suggests that Tregs play an important role in the control of allergy. AIT can drive the immune response towards induction of Treg cells resulting increased IL-10 and TGF-ß production and suppression of IgE production [30]. During AIT, the number of Th2 memory cells is reduced with an induced Treg/Th1 response [44]. Treg cells can be induced through high-dose allergen exposure, such as multiple stings received by beekeepers and prolonged, domestic cat-allergen exposure which have been demonstrated to induce IL-10-mediated tolerance and increased levels of antigen-specific IgG4. Furthermore, T cells producing IL-10 as well as Foxp3^+^IL-10^+^ T cells were shown to be increased in nasal mucosa of immunotherapy patients one [45]. IL-10 has an important role in the control of allergy by suppressing allergic inflammation and inducing regulatory T cells. The balance between allergen-specific T cell subsets may be influenced by AIT, as Tr1 cells have been found to be reduced in peripheral blood for atopic patients but increased in AIT treated patients [25•]. To summarize, Tregs appear to play an essential role in the induction of tolerance in AIT suppressing the allergic Th2 response and inducing the more tolerant Th1 state. Identifying their true relationship to clinical response remains to be investigated.

## B regulatory cells and immune tolerance

Regulatory B cells (Bregs) are a subset of CD19+ B cells that produce IL-10 and have the capacity to supress pro-inflammatory T and B cell effector function. Bregs promote immune tolerance state through production of interleukin-10 (IL-10), IL-35 and transforming growth factor b (TGF-β). IL-10-producing B cells have been most extensively studied for their regulatory potential in man and mice. To date, IL-10^+^ B cells have been reported to reside within CD1d^hi^CD5^+^, CD24^hi^CD27^+^, CD25^+^CD71^+^CD73^-^ and CD24^hi^CD38^hi^ B cell subsets [46••]. IL-10 producing CD25^+^CD71^+^CD73^-^ Breg subset has been shown to be induced following AIT. They regulate pro-allergic immune responses by inhibiting antigen-specific CD4^+^ T cell proliferation and produce anti-inflammatory IgG4 antibodies [46••]. Breg cells control excessive inflammatory responses through IL-10 secretion, and are involved in Treg cells differentiation by inhibiting release of proinflammatory cytokines [47•]. In bee-venom, tolerant individual, highly purified IL-10-secreting Breg cells were phenotypically characterized via high expression of surface CD25 and CD71 level but low level of CD73 (Human BR1 cells, CD73-CD25^+^CD71^+^ B cells) [46••]. In vitro studies revealed their capacity to suppress antigen-driven PBMCs proliferation. In the same study, bee-venom allergen (PLA2)-specific CD19^+^ B cells were increased following bee-venom AIT and produced IL-10 [46••].

## Functional IgG4 antibodies as potential biomarkers of AIT

An immunologic response following administration of large doses of the sensitizing allergen either by SCIT or SLIT has been associated with the induction of serum allergen-specific immune reactive and functional IgG antibodies (sIgG). A 10–100fold increase in the concentrations of allergen-specific IgG1 and, in particularly of IgG4 antibodies has been reported in several SCIT and SLIT studies [48, 49]. A correlation between serum allergen-specific IgG4 and clinical outcome measures have been explored in some but not all studies [50–53]. In a dose-response randomized double-blind placebo-controlled trial (RDBPCT) of SCIT, levels of sIgG4 were increased in a time- and dose-dependent manner [16••]. Conversely, allergen-IgE binding to B cells was decreased in a time- and dose-dependent manner. The serum inhibitory activity for IgE-FAB but not serum IgG4 correlated with combined symptoms and rescue medication scores (CSMS) at the population level but not at the individual level [16, 54•]. Furthermore, in a SCIT withdrawal study where participants were randomized to receive grass pollen-SCIT or placebo for 2 years, the actively treated group were further randomized to receive either placebo injections or SCIT for further 2 years. During treatment, the levels of sIgG1 and sIgG4 were shown to be increased at 2 years and at 4 years in the actively treated group. Those who received active treatment for 2 years and 2 years of placebo had increased levels of sIgG1 and sIgG4 at 2 years which declined (near 80%) at 4 years (2-year off treatment). Interestingly, serum inhibitory activity for IgE-facilitated allergen binding persisted 2 years after discontinuation of treatment. IgG4 depleted sera from patients who have discontinued immunotherapy showed reduction in the inhibitory activity of allergen-IgE binding. These findings indicate that functional and protective antibody responses is critical for long-term clinical tolerance [55••].

## Basophils and clinical responses to AIT

Basophils were originally identified by Paul Ehrlich in 1879. They comprise of <1% human leukocytes in peripheral blood. They contain cytoplasmic secretory granules, consisting of proteoglycans and histamine [56]. Basophils express FcεRI, which can be cross-linked by allergen-specific IgE after allergen exposure, resulting in degranulation with release of histamine, leukotrienes, and other mediators of the allergic inflammatory response [57, 58]. Surface expression of CD63 (granule-associated tetraspan) is typically detected on allergen-stimulated and activated basophils in whole blood, whereas CD203c is expressed on nearly all basophils regardless of their activation status. CD63 and CD203c (ectonucleotide pyrophosphatase/phospodiesterase 3, a type II transmembrane ectoenzyme) are complementary for assessing basophil activation [59, 60•]. Basophil also express multiple membrane proteins including CD13, CD107a, and CD164 which are expressed when activated. Furthermore, histamine can be measured in bronchoalveolar lavage fluid from those with allergic asthma and in plasma of atopic dermatitis patients [61]. More recently, intracellular expression of fluorochrome-labeled diamine oxidase (DAO) in basophils has been reported as potential novel biomarker of efficacy and tolerance after AIT [10••]. In a cross-sectional study of AIT, basophil activation induced by grass pollen allergen was elevated in seasonal allergic rhinitis (SAR) patients and diminished in SCIT or SLIT treated individuals. The reduction in basophil responsiveness and histamine release as measured by DAO using flow cytometry was associated with reduced combined symptoms and rescue medication scores [10••].

## Concluding remarks

**Fig. 2:**
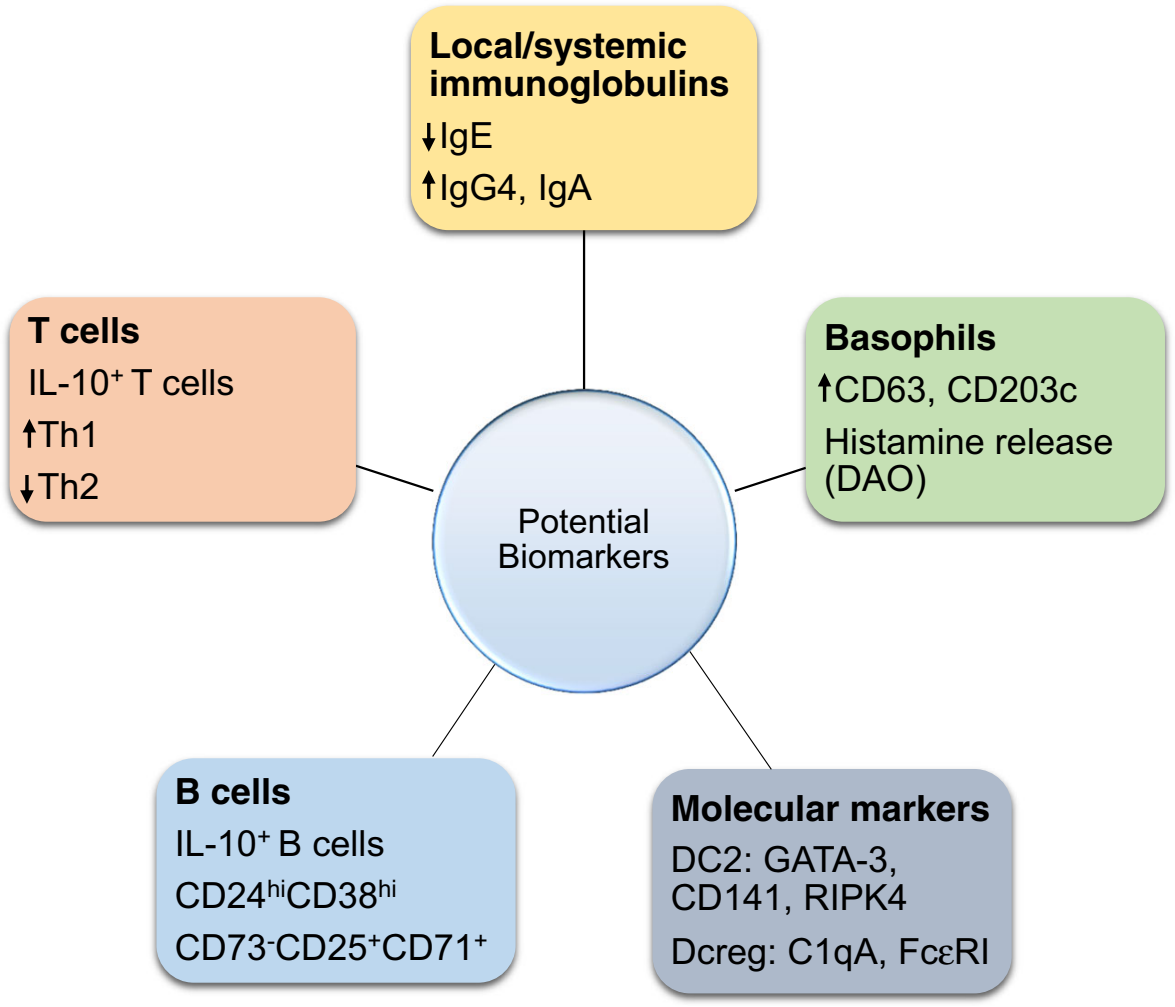
Biomarkers associated with allergen response and AIT. Biomarkers for various cell types may exhibit clinical benefit for allergen immunotherapy. The conventional T cell response to allergen mediating a CD4^+^ T cell response has been widely evaluated. The increase of CD4^+^ T cells in response to allergen elicits a Th2 cytokine storm such as IL-4, IL-5, and IL-13 causing an allergic response or asthma. On the other hand, Tregs have a protective role in allergy as they modulate immune tolerance to allergen and control the allergic reaction during allergen immunotherapy. AIT deviates the immune response towards Tregs increasing the IL-10 and TGF-ß production with a decrease in IgE, whereas the elevated IgG4 as well as IgA is mediated by B cells. The increase in Treg subsets Foxp3^+^ and Tr1 are also induced by AIT may be important biomarkers post-immunotherapy. The immune response can become resistant to immune deviation to Tregs/Th1 subsets; CD4^+^T cells (CD27-) expressing CRTH2 also produce a Th2 response. The CD27^-^ (Th2) subset have been found to be decreased in AIT treated subjects, whereas the protective CD27^+^ (Th2) subset was elevated. Additionally, IL10+ Bregs can induce protective IgG4 response and inhibit the antigen-specific CD4^+^ T cell proliferation during AIT. Furthermore, the DC markers in response to immunotherapy have an increased DCreg (C1QA and FcεRIIIA) and decreased DC2 (GATA-3, CD141, and RIPK4) response. A novel biomarker for basophils has also been established intracellular expression of fluorochrome-labeled diamine oxidase, which was increased in SAR patients but reduced during SCIT and SLIT. The reduction in histamine, CD63, and CD203c by basophils was also associated with reduced symptoms.

Our current understanding of the application of AIT has highlighted a series of possible immunological biomarkers which can be utilized to determine efficacy of treatment efficacy (Fig. 2). It is known that both innate and adaptive immune responses are modulated during AIT. The reduction of ILC2s in combination with Tregs and Bregs result in skewing towards a Th1. IgG4 antibodies have the capacity to inhibit both allergen-induced basophil responsiveness and CD23-mediated IgE-facilitated allergen presentation. Although these changes are observed repeatedly after AIT, identifying their true relationship to clinical response remains to be investigated before they can be applicable as surrogate biomarkers. Functional IgG4, represented by IgE-FAB, however, appears to be a promising biomarker with correlation with clinical outcome as well as tolerance. Molecular markers identified in DC2 (CD141, GATA3, and RIPK4) and DCreg (C1QA and FcγRIIIA) induced by AIT are crucial emerging biomarkers for clinical response. There is an urgent need for studies correlating the knowledge on immunological changes in AIT before biomarkers can play a role in personalized medicine.
